# A New Treatment of Tapeworm

**Published:** 1878-10

**Authors:** 


A New Treatment of Tapeworm.—From the results of numerous experiments, M. Bouchut had ascertained that not only ascarides, but fragments of tænia, when placed in a weak alcoholic solution containing one thirty-fifth of amylaceous pepsine, are digested by the fluid in the course of twelve hours. We thus obtain an artificial digestion of the animal matter exactly similar to that which ensues when meat is treated by the same process. On submitting the conclusion drawn from his experiments to the test of practice at the Enfans Malades, M. Bouchut found that the solution of pepsine was eminently successful. If his experience be confirmed, a valuable addition will be made to adult as well as infantile therapeutics. In conclusion, we may observe that animal food is, almost certainly, the channel through which the parasite is conveyed; and hence that official inspection of suspected dealers in meat would form a useful adjunct to the practice of the physician.—*Louisville Medical News.*

